# How to predict seasonal weather and monsoons with radionuclide monitoring

**DOI:** 10.1038/s41598-019-39664-7

**Published:** 2019-02-25

**Authors:** Lucrezia Terzi, Martin Kalinowski, Michael Schoeppner, Gerhard Wotawa

**Affiliations:** 1 Belgian Nuclear Research Centre (SCK•CEN), Mol, Belgium; 2 Technische Universität Wien, Atominstitut, Austria; 3 Provisional Technical Secretariat, Preparatory Commission for the Nuclear-Test-Ban Treaty Organization, Vienna, Austria; 4 Institute of Safety/Security and Risk Sciences, University of Natural Resources and Life Sciences, Vienna, Austria; 5 Zentralanstalt für Meteorologie und Geodynamik (ZAMG), Vienna, Austria

## Abstract

Monsoon in India is of particular importance for the $2 trillion economy, highly dependent on agriculture. Monsoon rains water two-thirds of India’s harvest. However, the monsoon season also causes large-scale flooding, resulting in loss of human life and economic damage estimated around $7 billion annually. Beryllium-7 is a tracer that can be used to monitor the intensity of stratosphere-troposphere exchange, which varies in accordance with the annual cycle of the global atmospheric circulation (Hadley, Ferrel and Polar cells). Based on the beryllium-7 data collected globally as part of the monitoring of the Comprehensive Nuclear-Test-Ban Treaty, the presented empirical method demonstrates the possibility to predict the start, withdrawal and intensity of the Indian monsoon season. Onset can be forecasted with an unprecedented accuracy of ±3 days, 2 months in advance compared to 1–3 weeks in advance by traditional methods. Applying this new method will enable better preparation for economic and natural hazard impacts of the monsoon season in India. This method can also be extended to other regions where the movement of Hadley cells governs monsoon onset and withdrawal.

## Introduction

**Figure 1:**
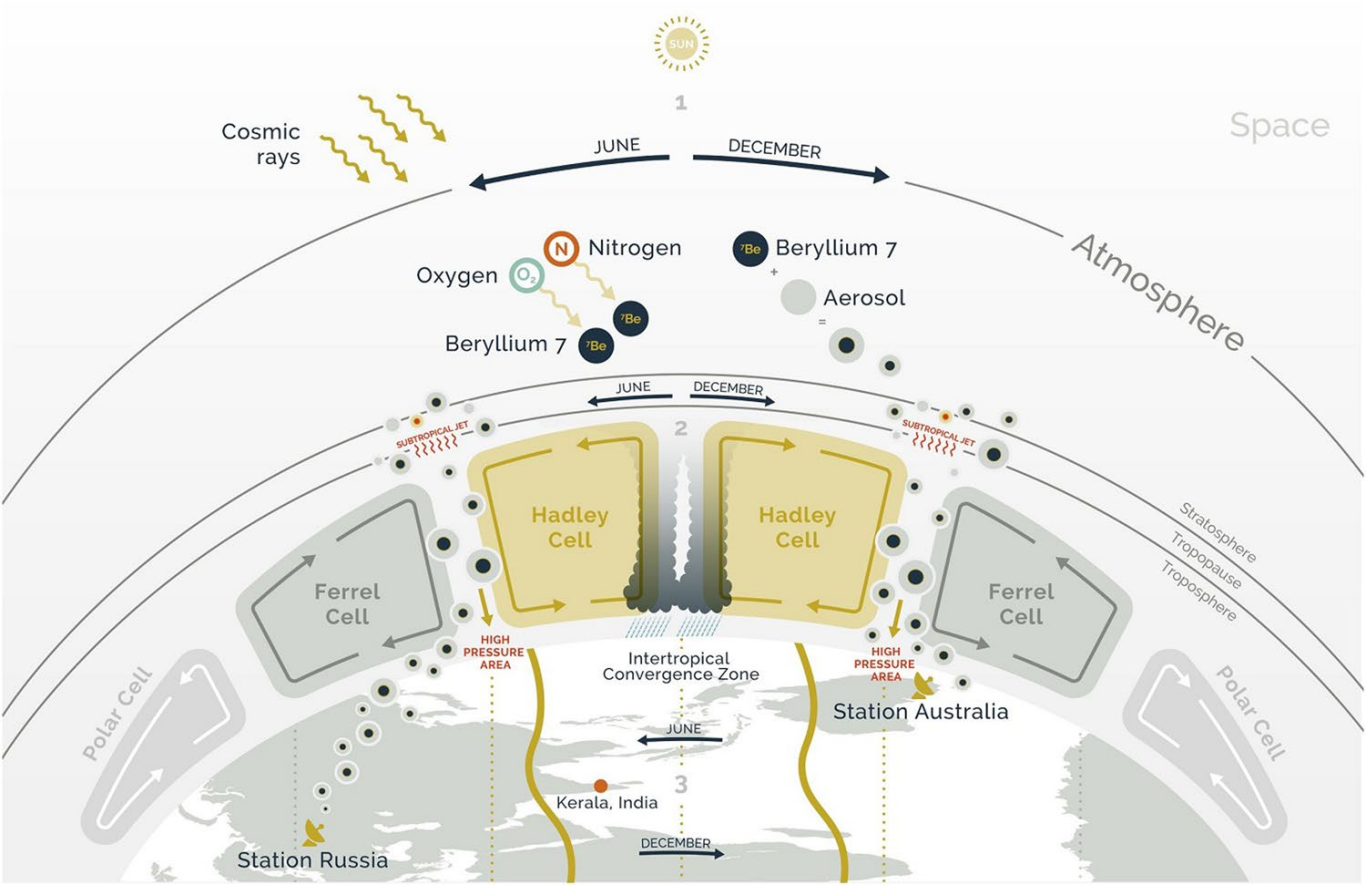
Points 1, 2, and 3 show the link between the heat produced by sunlight, earth rotation and Hadley cell progression with the monsoon pathway delimited by the intertropical convergence zone. Upper part: origination of beryllium-7. It is created through spallation from cosmic rays at high altitudes, attaches to aerosols and becomes detectable at monitoring stations on the earth’s surface after moving downwards with air masses due to atmospheric cell cycles. The vertical flux of air is going downward bringing greater quantities of fresh beryllium-7 toward the station. Lower part: the inter tropical convergence zone (ITCZ) follows the sun moving northward from January to June and southward from July to December. As the sun moves, the ITCZ moves and consequently also the Hadley-Ferrel and Polar cells. If a station is right below the ITCZ, the detected concentration of beryllium-7 will be low because the amount of beryllium-7 brought toward the station is washed out by rain and cannot be measured. Moreover, at ITCZ the vertical air flux is going upward so no fresh beryllium-7 reaches the surface where the station is located. If a station is right below the Hadley-Ferrel convergence zone (HFCZ), the concentration of beryllium-7 is high because the vertical flux of air is going downward bringing greater quantities of fresh beryllium-7 toward the station. Red dot indicates the region of Kerala in India, used as reference point of the Indian monsoon. High pressure area (red marked) indicates the surface location of high-pressure ridge formed in coincidence with the subtropical jet stream as a result of the Hadley circulation. mrprezident or www.mrprezident.com.

Beryllium-7 is one of the isotopes regularly detected by the radionuclide monitoring network of the Comprehensive Nuclear-Test-Ban Treaty Organization (CTBTO)^1^. Beryllium-7 is naturally produced in the upper troposphere and lower stratosphere, where it is formed by spallation of nitrogen and oxygen from high-energy cosmic particles (Fig. 1)^2,3^. Its half-life of 53.2 days is long enough to make it a suitable tracer of the air mass exchange between stratosphere and troposphere^4^. An increase in beryllium-7 at the surface level is an indicator of a vertical downward flux of air masses^5–8^. Thus, a general increase or decrease of its concentration over time can be utilized as a proxy to monitor seasonal movement and general dynamics of atmospheric cells such as the Hadley-Ferrel Convergence Zones (HFCZ)^9–11^. Many CTBTO monitoring stations exhibit seasonal patterns in their concentrations of beryllium-7 and other isotopes, potentially bearing even more correlations with weather phenomena other than the ones discussed in this report^9^. The close relationship between the position of the HFCZ and the monsoon as demonstrated in this study makes beryllium-7 a reliable indicator of monsoon onset and withdrawal forecasts. It is a more reliable index than water content as it is linked to the root cause rather than the effects resulting from monsoon phenomena. Cross correlations of beryllium-7 activity concentrations at different monitoring stations can thus potentially serve as a suitable early warning index in monsoon regions governed by the HFCZ movements, with potentially several months of lead time prior to monsoon onset and withdrawal as demonstrated in the case study below on the Indian Monsoon.

Monsoons are governed by the periodic seasonal movement of the Intertropical Convergence Zone (ITCZ)^12–15^. They are associated with periods of frequent and intense precipitation, typically lasting a few months, preceded and followed by dry and stable weather conditions. They are linked to the trade winds, influenced by earth’s rotation and intense solar heating, and associated with deep convection in the equatorial region, which activates the Hadley circulation. As air masses over land, compared to those over the sea, heat up faster with the beginning of summer, moist air first moves inland, which loses its water content while rising (Fig. 1). The air then moves back seaward at higher altitudes. Seasonal variations of the Hadley circulation due to the tilt of the earth’s axis are the driving force for the world’s monsoons. Monsoon regions are located in Asia-Australia, West Africa, and to a lesser degree in North and South America^16^. Important monsoon parameters are onset, withdrawal, and intensity as they strongly influence agriculture, water management, emergency planning, public health, and logistics^17,18^. Originally, monsoon forecasts were based on statistical models of local measured variables. Today, most of the short-term monsoon predictions are based on numerical weather prediction or taking local meteorological parameters as indicators. Our new method for monsoon prediction is based on the concentration of beryllium-7 in the atmosphere near ground level at specific locations outside the monsoon area. The ground-level concentrations are strongly influenced by the inward convection of the HFCZ directly connected to the Hadley circulation and therefore to the monsoon formation mechanism (Fig. 1). Unlike using statistics of local parameters where monsoon is expected, beryllium-7 concentration timeseries from two monitoring sites, north and south of the equator, are required. These timeseries show a seasonal pattern directly linked to the Hadley circulation. Their change over time indicates the progression of atmospheric cells, which governs the monsoon circulation. The effect of the Hadley-Ferrel downward convection on selected timeseries proves, within the limited dataset of only 10–15 years, to be a reliable indicator of Hadley circulations. The capability to measure the downward flux of an atmospheric cell allows to determine the approach of the monsoon onset.

Atmospheric beryllium-7 concentration time series from near-surface measurements are analyzed in this study for correlations with monsoon onset and withdrawal over a ten-year period. The statistical significance and the methodology are limited to the length of the available dataset of beryllium-7. Long-term significance of the findings will be demonstrated with time and larger datasets which are currently not available.

While the presented study focuses on the Indian monsoon, the transferability of this method to other monsoon regions has been tested in case studies. As long as the monsoon is triggered by progression of atmospheric cells such as the Hadley cell for the monsoon over Kerala (southwest India), the trans-equatorial method can be applied to other monsoon regions as well^19^.

## Case study on the Indian Monsoon

**Figure 2:**
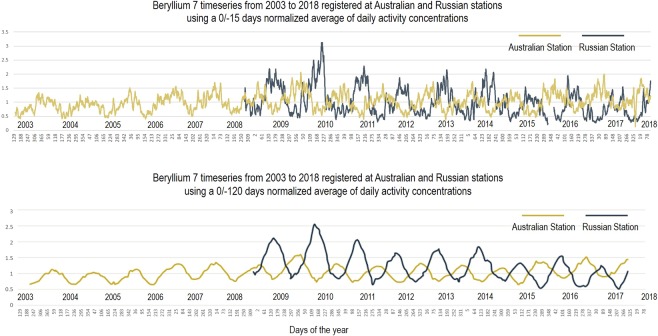
Multi-year time series (2003–2018) from Australian (orange line) and Russian Stations (blue line) based on normalized daily activity concentration of beryllium-7 averaged over 0/−15 days and 0/−120 days. Each data point represents the average of the past 15 days (top chart) and of the past 4 months (lower chart).

Summer monsoon in Kerala, India is well researched and documented. Based on historical average, the Indian Meteorological Department (IMD) assigned the normal monsoon onset date to June 1^st^, and announces its onset forecast in the mid of May. Monsoon onset is defined by the IMD as change in rainfall, wind field, and Outgoing Longwave Radiation (OLR). Given that the monsoon onset moves in a time window of 25 days according to the IMD onset dates since 1980, the warning time currently provided is between 1–3 weeks with an error margin of ±5 days^19^. The Indian monsoon over Kerala is used here to explain and demonstrate our novel forecast method based on beryllium-7. The map in Fig. 1 shows the two CTBTO stations (one in Dubna, Russia and the other one in Melbourne, Australia) selected to make seasonal predictions for the Kerala monsoon. Beryllium-7 measured at these locations is influenced by the seasonal movement of the ITCZ. Thus, the moving average of the concentrations at each station (Fig. 2) follows at first approximation a sine-like curve with a period of one year. Beryllium-7 timeseries are built as normalized moving average of past 15 days (0/−15) activity concentrations. Every normalized value represents the average concentration of past 15 days, which is then normalized by dividing each averaged value by all available historical data of that specific timeseries. The primary purpose of moving averages is to smooth out short-term variations due to local processes such as wash-out by rain^6^ and cyclonic events. The type of statistical calculation such as moving average and normalized moving average are applied to increase data accuracy. Appropriate timeseries trendline calculations (normalization and period of moving averages) may differ on the basis of station, monsoon region, and type of analysis needed. For instance, seasonal shift calculations and atmospheric cell progression trendlines are built using a moving average with a period of 4 months (0/−120 days) (Fig. 2, lower chart).

## Trans-equatorial Approach

Historically, several methods have been applied to study the monsoon phenomena. These methods are usually based on different indicators or a combination of indicators such as precipitation, moisture, and winds transition^20–26^. The trans-equatorial approach, as presented here, is based on determining the temporal tipping point of the monsoon, not in terms of temperature and humidity^27^, but in terms of beryllium-7 concentrations. This method looks at the seasonality of radioisotopes’ concentrations along the high-pressure ridge, also called subtropical high belt. It is continuous in both hemispheres and the result of the subtropical jet stream formed by the HFCZ^5^. All stations located under the high-pressure ridge can be used for determination and prediction of the monsoon tipping point.

The value of any monsoon onset prediction method is determined by its reliability to forecast accurate dates and the length of its lead time. Every time the trendlines of the Russian and Australian stations cross each other, that particular date is referred to as a cross point. There are two cross points per year: CP1 in early spring and CP2 in late summer. The number of days from CP1 to the monsoon onset is the forecast lead time. By averaging the lead time from CP1 over the past 10 years, an almost constant alerting time for the monsoon onset of 52 ± 3 days is achieved. After the monsoon onset date has passed, the second cross point (CP2) occurs between the two stations on average 42 ± 7 days before the monsoon withdrawal date, representing the monsoon withdrawal lead time. All cross points are correlated with monsoon onset and withdrawal times because the seasonal movement of the Hadley-Ferrel Convergence Zone is coherent with the Hadley circulation, which drives the monsoons in and out of India.

Monsoon onset and withdrawal forecasts are built by adding the constant lead time obtained using a multi-year average to the date when either CP1 or CP2 occur. For example, for normalized average of the past 15 days, the onset lead time of 52 days is added to the day CP1 occurs. The correlation between onset and withdrawal versus their cross points (CP1 and CP2) is 0.89 and 0.73 respectively. The beryllium-7 cross point technique has been compared with the change point (CHP) index method confirming improved accuracy and extended onset lead time for all available years^20,28^.

As shown in the past decade, there is a direct correlation between monsoon length and intensity of Monsoon. IMD defines monsoon intensity as percentage of Long Period Average (LPA) of the rainfall over the country as a whole during the monsoon season from June to September. Since the length of the monsoon can be determined with this method, conclusions about its intensity can also be made. The further apart CP1 and CP2 are, the longer and more intense the monsoon will be.

This transequatorial approach has been developed to make monsoon onset predictions as early, reliable, and accurate as possible over time.

**Figure 3:**
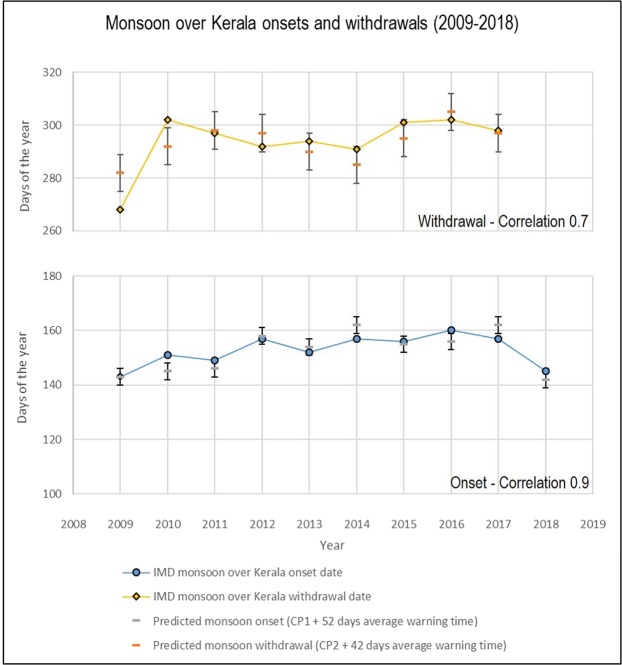
Forecast for 2009–2018 monsoon onset over Kerala using transequatorial approach. Onset prediction is based on a lead time of 52 ± 3.7 days; withdrawal prediction on a lead time of 42 ± 7.3 days. Correlation between IMD monsoon onset and predicted onset using transequatorial method (CP1 + averaged lead time) is 0.89. Correlation between IMD monsoon withdrawal and predicted withdrawal using transequatorial method (CP2 + averaged lead time) is 0.73.

The presented method uses CP1 for monsoon onset and CP2 for monsoon withdrawal where trendlines are built as normalized moving average of the past 15 days. Figure 3 show the results: onset is predicted with 52 days lead time and a precision of ±3.7 days, while withdrawal is predicted with 42 days lead time and a standard deviation of ±7.3.

## Data and Predictions for 2018

For 2018, the predicted onset using the beryllium-7 trans-equatorial method is in accordance with the forecasted monsoon from IMD and the actual onset date: a monsoon of normal intensity with an earlier onset than the normal date, i.e. before June 1^st^. Later, after the monsoon has started, IMD reported the onset to have started in the south of Kerala by 25^th^ May moving towards the northern border by the 28^th^ of May. The transequatorial approach has been applied to determine the monsoon onset date for 2018. Figure 3 shows the date obtained by CP1 plus its multi-year average lead time for 2018 onset prediction.

Based on a normalized moving average of past 15 days, CP1 in 2018 occurred on day #90; the average monsoon lead time is 52 days. Therefore, the forecasted onset date is day #142 (22^nd^ May), only 3 days before from the actual onset date for 2018.

Further comparison with onset derived from objective and standardized indices such as OCI and HOW1 is necessary^29–31^.

With an average monsoon withdrawal date on day #294 (21^st^ of October), the monsoon is predicted to be more than a week longer and with higher intensity than last year.

In order to fill the gaps between points in time for the presented method, additional locations for cosmogenic radionuclide detection would be necessary or other statistical features than the cross points of two beryllium-7 timeseries could be utilized.

The current acquisition time for International Monitoring System (IMS) radionuclide samples is three days. Therefore, in view of real time monsoon early warning, three additional days need to be considered for the calculation of beryllium-7 activity concentrations and included in warning time lags.

## Conclusion

The new beryllium-7 trans-equatorial method as introduced here offers a simple and useful mechanism to predict seasonal weather such as the formation of monsoons triggered by HFCZ progression^28^. The method allows longer forecast lead times and improved accuracy compared with traditional methods currently utilized. This provides significantly improved planning opportunities for agriculture, water management, emergency management, public health management, and logistics.

Our results demonstrate that beryllium-7 is a suitable tracer for many atmospheric phenomena related to stratosphere-troposphere exchange, and its monitoring can be used to obtain an improved understanding of the global atmospheric circulation and its inter-annual variability. The method described here has high potential to improve annual monsoon forecasts in the future, especially as confidence will increase with additional datasets becoming available.

### Disclaimer

The views expressed on this paper are those of the authors and do not necessarily reflect the views of SCKCEN, ZAMG, BOKU or CTBTO Preparatory Commission.

## Supplementary information

Supplementary information

Dataset 1

## Data Availability

The data that were used for the current study and that support its findings are not publicly available, but access through the virtual Data Exploration Centre (vDEC) can be granted by the CTBTO via a cost-free confidentiality agreement. The application for vDEC access to data can be submitted for approval through a simple web form at https://www.ctbto.org/specials/vdec/.”
