# Supplementary material for: How to predict seasonal weather and monsoons with radionuclide monitoring
Source: Sci Rep. 2019 Feb 25;9:2729. doi: 10.1038/s41598-019-39664-7 (PMC6390104; doi:10.1038/s41598-019-39664-7)
Supplement: Supplementary file 1 — Supplementary information [file 41598_2019_39664_MOESM1_ESM.pdf]

Extended Data

Title: How to predict seasonal weather and monsoons with radionuclide monitoring

Authors: Lucrezia Terzi [a,b,*], Martin Kalinowski [c], Michael Schoeppner [c, d] ,Gerhard Wotawa [e]

Affiliations: [a] Belgian Nuclear Research Centre (SCK•CEN), Mol, Belgium and [b] Technische Universität Wien, Atominstitut, Austria; [c] Provisional Technical Secretariat, Preparatory Commission for the Nuclear-Test-Ban Treaty Organization, Vienna, Austria; [d] Institute of Safety/Security and Risk Sciences, Vienna, Austria; [e] Zentralanstalt für Meteorologie und Geodynamik (ZAMG), Vienna, Austria.

***Corresponding Author:**

Lucrezia Terzi

Ph: +43 6644553833

e-mail: lucrezia.terzi@sckcen.be

[a] SCK•CEN, Belgium Nuclear Research Centre,

Boeretang 200, 2400 Mol, Belgium.

[b] Technische Universität Wien, Atominstitut,

Stadionallee 2, 1020 Wien, Austria.

Supplementary Tables

Table S1. **For each year the cross-point day # and predicted monsoon onset day # is given based on ^{7}Be trans-equatorial approach. Three methods with different warning times: Warning time based on all available years (2009-2017), Warning time based on 2009-2013 and applied to 2014-2018, Warning time based on all previous years average, which is the prediction time that develops with each year. For each predicted monsoon onset provided the corresponding difference from IMD monsoon onset is given. Kerala is the reference region for monsoon onset. Accuracy (trueness and precision) is defined by ISO 5725-1.**

Transequatorial approach - Onset											
			Warning time based on all available years			Warning time based on 2009-2013 and applied to 2014-2018			Warning time based on all previous years		
Year	IMD monsoon over Kerala onset date	cross point 1 (CP1)	52 days average warning time based on all available years (2009-2017)	Predicted monsoon onset (CP1 + 52 days average warning time)	Difference between IMD monsoon onset and predicted onset (n. of days)	53.2 days average warning time based on 5 years (2009-2013)	Predicted monsoon onset (CP1 + 53.2 days average warning time)	Difference between IMD monsoon onset and predicted onset (n. of days) based on 53.2 days lead time	Average warning time based on previous years	Predicted monsoon onset (CP1 + previous years average warning time)	Difference between IMD monsoon onset and predicted onset (n. of days) based on previous years average lead time
2009	143	91	52	143	0	52					
2010	151	93	58	145	-6	58			52	145	-6
2011	149	94	55	146	-3	55			55	149	0
2012	157	106	51	158	1	51			55	161	4
2013	152	102	50	154	2	50			54	156	4
2014	157	110	47	162	5	47	163.2	6.2	53	163	6.2
2015	156	103	53	155	-1	53	156.2	0.2	52	155	-0.83
2016	160	104	56	156	-4	56	157.2	-2.8	52	156	-3.71
2017	157	110	47	162	5	47	163.2	6.2	53	163	5.75
2018	145	90	55	142	-3	55	143.2	-1.8	52	142	-2.89
Correlation between IMD monsoon onset and predicted onset using transequatorial method (CP1 + averaged warning time)			0.89			0.86			0.84		

Accuracy	Trueness- Difference between IMD monsoon onset and predicted onset using transequatorial approach	-0.39	1.60	0.72
	Precision- Standard deviation of the Difference between IMD monsoon onset and predicted onset using transequatorial approach	3.7	4.34	4.44

Table S2. **For each predicted monsoon withdrawal provided using transequatorial approach (CP2 obtained with 0/-15 days normalized average of ^{7}Be activity concentrations) the corresponding difference from IMD monsoon withdrawal is given. Three methods with different warning times: Warning time based on all available years (2009-2017), Warning time based on 2009-2013 and applied to 2014-2017, Warning time based on all previous years average, which is the prediction time that develops with each year. With warning time based on all previous years, due to the increased weight of 2010 offset, withdrawal present results in a correlation of 0.23 versus 0.90 obtained when 2010 offset is excluded. Accuracy (trueness and precision) is defined by ISO 5725-1. Kerala is the reference region for monsoon withdrawal.**

Transequatorial approach - Withdrawal											
			Warning time based on all available years			Warning time based on 2009-2013 and applied to 2014-2018			Warning time based on all previous years		
Year	IMD monsoon over Kerala withdrawal date	cross point 2 (CP2)	42 days average warning time based on all available years (2009-2017)	Predicted monsoon withdrawal (CP2 + 42 days average warning time)	Difference between IMD monsoon withdrawal and predicted monsoon withdrawal (n. of days)	40.8 days average warning time based on 5 years (2009-2013)	Predicted monsoon withdrawal (CP2 + 40.8 days average warning time)	Difference between IMD monsoon withdrawal and predicted withdrawal (n. of days) based on 40.8 days lead time	Average warning time based on previous years	Predicted monsoon withdrawal (CP2 + previous years average warning time)	Difference between IMD monsoon withdrawal and predicted withdrawal (n. of days) based on previous years average warning time
2009	268	240	28	282	14	28					
2010	302	250	52	292	-10	52			28	278	-24
2011	297	256	41	298	1	41			40	296	-1
2012	292	255	37	297	5	37			40	295	3.33
2013	294	248	46	290	-4	46			40	288	-6.5
2014	291	243	48	285	-6	48	283.8	-7.2	41	284	-7.2
2015	301	253	48	295	-6	48	293.8	-7.2	42	295	-6.00
2016	302	263	39	305	3	39	303.8	1.8	43	306	3.86
2017	298	255	43	297	-1	43	295.8	-2.2	42	297	-0.63
2018	n/a*	n/a*		-	-	-	-	-	-	-	-
Correlation between IMD monsoon withdrawal and predicted withdrawal using transequatorial method (CP2 + averaged warning time)			0.73			0.90			0.23		

Accuracy	Trueness-<br>Difference between IMD monsoon withdrawal and predicted withdrawal using transequatorial approach	-0.44	-3.70	-4.77
	Precision-<br>Standard deviation of the Difference between IMD monsoon withdrawal and predicted withdrawal using transequatorial approach [n. of days]	7.23	4.36	8.88

*data not available at time of submission

Supplementary Figures

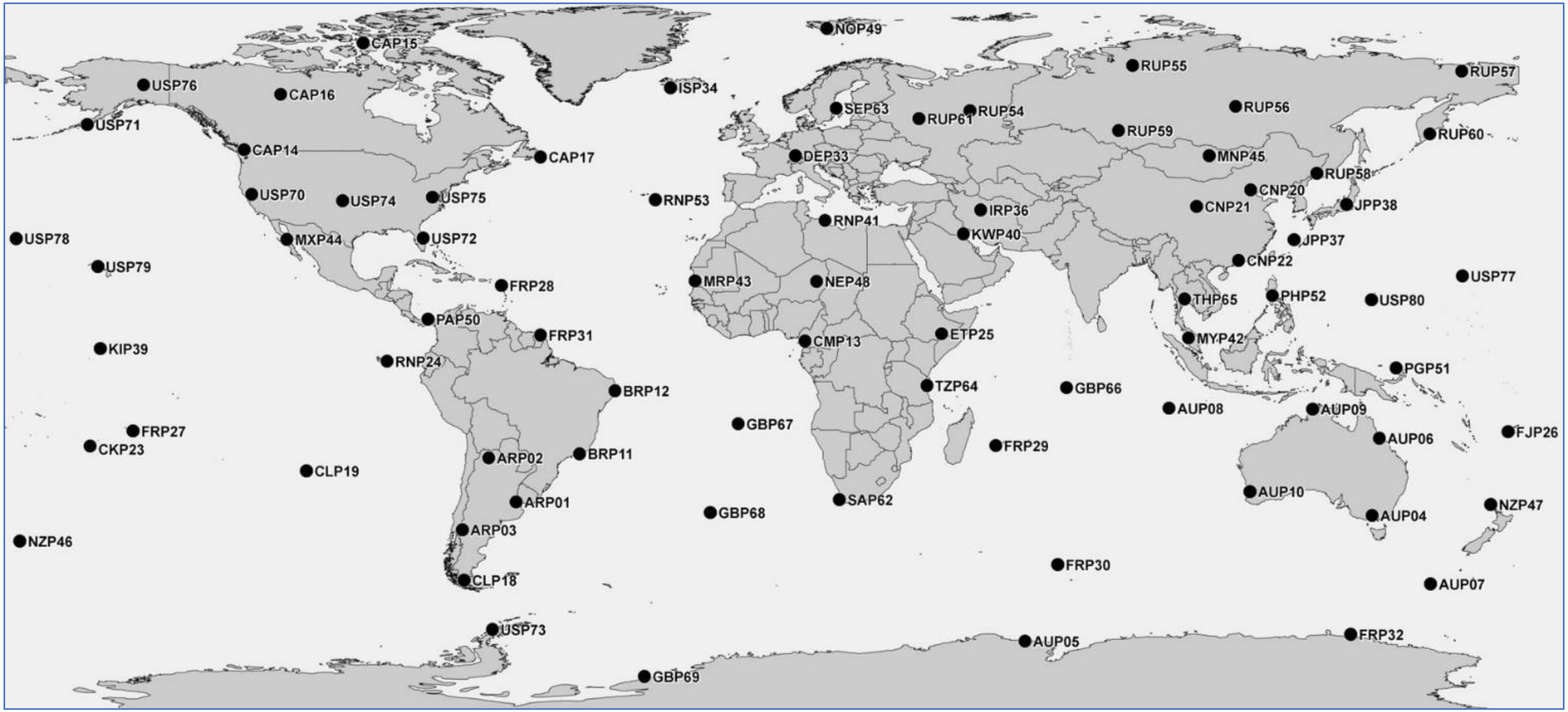

Figure S1. **The radionuclide component of the International Monitoring System consists of 80 stations (70 stations currently certified for operation) and aims at the detection of signature isotopes from nuclear explosions.**

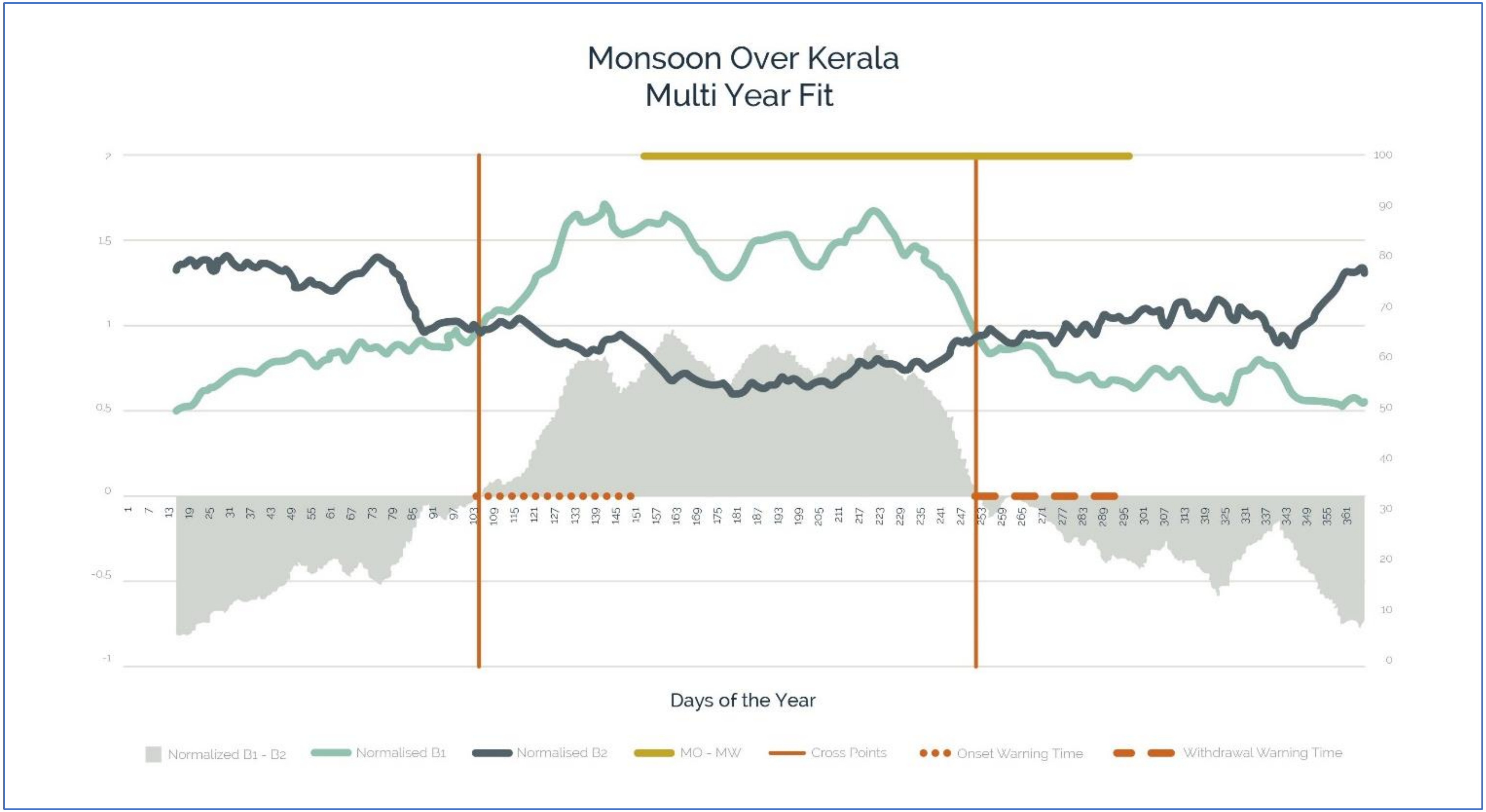

Figure S2. Multiyear-average plot of beryllium-7 activity concentrations from 2009 to 2018. Beryllium-7 (*^{7}Be)* [μBq/m³] timeseries from Russian (B1 – light green line) and Australian (B2- dark blue line) stations are averaged using 0/-15-day average and normalized with the overall average value of each timeseries. Cross points 1 (CP1) and cross point 2 (CP2) are marked with red bars, their provided warning times on onset (52 days) and withdrawal (42 days) are marked with red dotted lines. Monsoon period is shown with yellow bar on top of the chart. Onset and withdrawal dates of monsoon over Kerala are retrieved by the Indian Meteorological Institute.

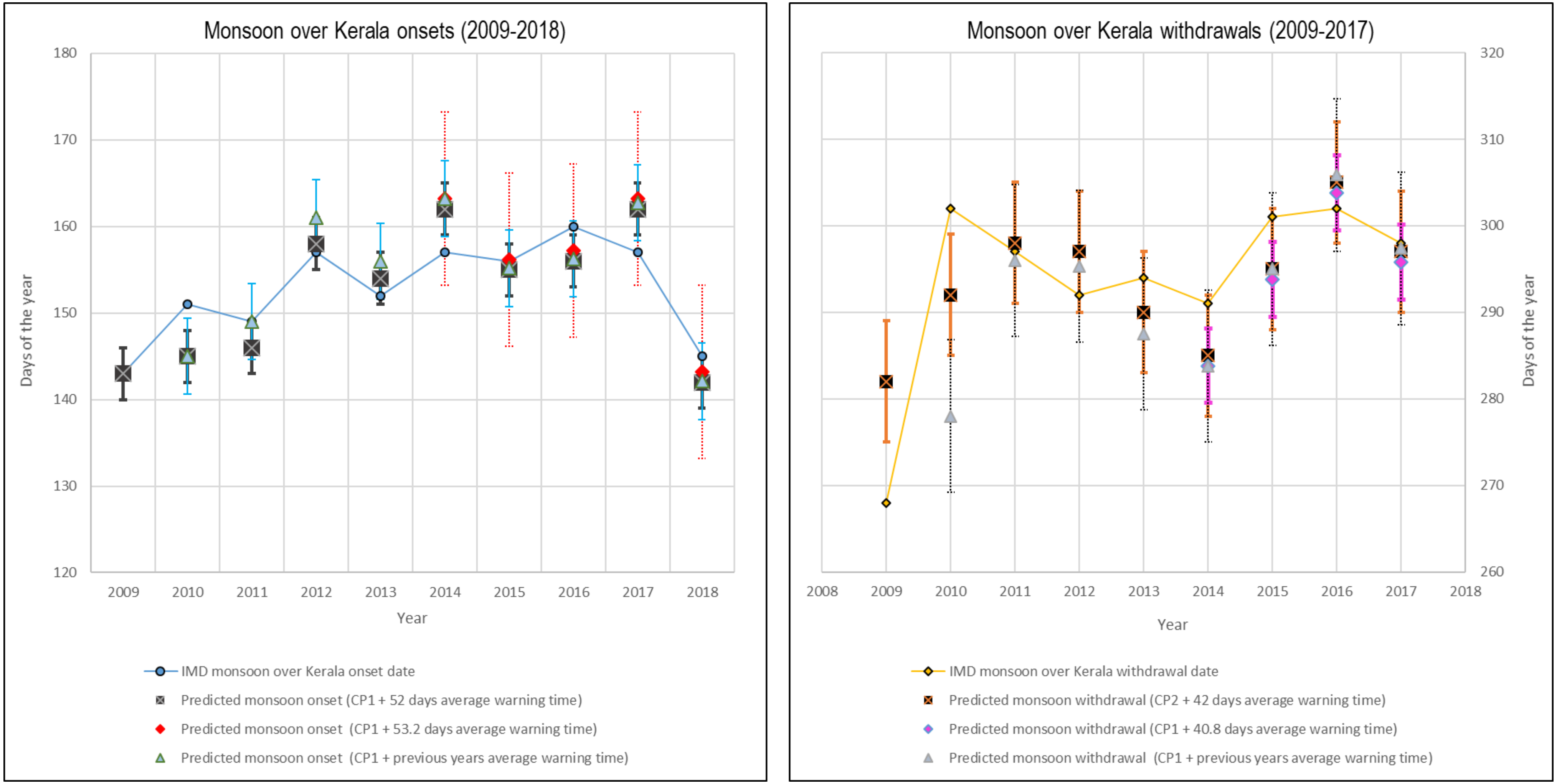

Figure S3. **IMD monsoon onset dates (blue dots with straight line) and IMD monsoon withdrawal dates (orange diamonds with straight line) versus their respective predicted arrivals calculated using transequatorial approach. Three predicted onset and withdrawal dates are plotted depending on how the averaged lead time is calculated: averaged lead time based on all available years (2009-2017) (crossed square), average lead time based on split dataset (2009-2013) (diamonds), average lead time based on previous years, which is the prediction time that develops with each year(triangles). Cross points are obtained over normalized 0/-15 days averaged ^{7}Be activity concentration values. Kerala is the reference region for monsoon onset.**

Supplementary Formulas

***Formula S1*. (1) Mean calculation and (2) normalized mean calculation used for trans equatorial approach. Where x^i is the ^{7}Be activity concentration recorded each day [µBq/m^3]. *N* is the number of days for which the mean is calculated: 0/-15 for the presented method. *M* is the calculated mean. $\bar{x}$ is the overall averaged activity concentration of ^{7}Be [µBq/m^3] measured at each location: 2003-2018 for Melbourne, Australia and 2009-2018 for Dubna, Russia. *N(M)* is the calculated normalized mean.**

$$M = \frac{1}{n} * \sum_{i=1}^{n} x^i \quad (1)$$

$$N(M) = \frac{1}{\bar{x}} * \frac{1}{n} * \sum_{i=1}^{n} x^i \quad (2)$$
